# PPARγ Mediates the Cardioprotective Roles of Danlou Tablet After Acute Myocardial Ischemia-Reperfusion Injury

**DOI:** 10.3389/fcvm.2022.858909

**Published:** 2022-03-25

**Authors:** Meng Wei, Mengying Guo, Xinxiu Meng, Lin Li, Hongyun Wang, Mingxue Zhang, Yihua Bei

**Affiliations:** ^1^Cardiac Regeneration and Ageing Lab, Institute of Geriatrics (Shanghai University), Affiliated Nantong Hospital of Shanghai University (The Sixth People's Hospital of Nantong), School of Medicine, Shanghai University, Nantong, China; ^2^Shanghai Engineering Research Center of Organ Repair, School of Life Science, Shanghai University, Shanghai, China; ^3^Affiliated Hospital of Liaoning University of Traditional Chinese Medicine, Shenyang, China

**Keywords:** Danlou tablet, PPARγ, ischemia-reperfusion injury, cardiomyocyte, apoptosis

## Abstract

Ischemic heart disease is one of the biggest threats to human life in the world. Reperfusion therapy is an effective strategy to reduce infarct size and ischemic injury. However, reperfusion process may cause secondary myocardial injury which is defined as ischemia-reperfusion injury (IRI). Exploring potential therapeutic strategy to attenuate IRI is extremely important. Danlou tablet (Dan), a Chinese herbal compound consisting of ten herbs, has been identified to be protective for the heart. However, the mechanism of Dan-induced cardioprotection after acute reperfusion was unelucidated. In this study, to investigate the role and mechanism of Dan in myocardial IRI, we performed acute IRI modeling in mice and oxygen-glucose deprivation–reperfusion (OGD/R)-induced apoptosis in primary neonatal rat cardiomyocytes (NRCMs). We found that Dan had protective effect against acute IRI in mice, as evidenced by reduced infarct size, TUNEL-positive cardiomyocytes (CMs), and Bax/Bcl2 ratio and cleaved-caspase 3/caspase 3 ratio *in vivo*. Meanwhile, Dan inhibited OGD/R-induced apoptosis of NRCMs *in vitro*. Mechanistically, Dan could activate proliferator-activated receptor gamma (PPARγ) in both IRI hearts and OGD/R-stressed NRCMs, while inhibition of PPARγ attenuated the protective effect of Dan against IRI *in vivo* and OGD/R-induced CM apoptosis *in vitro*. These data reveal that Dan attenuates acute myocardial IRI and CM apoptosis through activating PPARγ. Our findings may extend the knowledge of Chinese medicine and provide potential strategy for the precise treatment of ischemic heart diseases.

## Introduction

Cardiovascular disease (CVD), especially ischemic heart disease, is one of the biggest killers to human life worldwide (1, 2). Percutaneous coronary intervention (PCI)–reperfusion therapy is an effective strategy to rescue ischemic injury and to reduce the risk of death (3, 4). Nevertheless, reperfusion can cause secondary myocardial injury in the patients undergoing PCI therapy (5). This sudden myocardial reperfusion after ischemia can induce a series of pathological processes, such as oxidative stress, Ca^2+^ overload, altered mitochondrial function, DNA strand breaks, and cell damages, which is termed as ischemia-reperfusion injury (IRI) (6–8). Prolonged myocardial IRI may further develop cardiac remodeling and even heart failure, which severely influences clinical prognosis (9).

Ischemia-reperfusion injury-induced cardiomyocyte (CM) apoptosis has been attracting an increasing attention in the past years, and exploring potential therapeutic medicine to rescue IRI is extremely important. To date, several potential molecules or targets are identified effective to attenuate IRI. For example, exendin-4, a glucagon-like protein-1 receptor agonist, was found to protect myocardium against IRI in rats (10). Noncoding RNAs, microRNA (miR)-486, was reported to be downregulated upon IRI, while increasing miR-486 can relieve IRI and myocardial apoptosis (11). Long noncoding RNA (lncRNA) CPhar was identified to be induced by exercise training, whose upregulation can protect against IRI (12). Recently, it was reported that inhibition of acid sensing ion channel 1a can recover cardiac function after IRI (13). Absolutely, increasing studies have been performed to explore an appropriate method to protect the heart against IRI. However, the potential role and biological mechanism of Chinese traditional medicine is poorly explored in IRI therapy.

Danlou tablet (Dan), a Chinese proprietary medicine, has been used for angina pectoris treatment (14). From 2012, scientists and doctors tried to investigate the protective roles of Dan in heart. It was found that Dan can improve cardiac function in swine with coronary disease (15). Later, Dan was identified effective to attenuate arrhythmia in rats (16), peri-procedural myocardial injury (17), and atherosclerosis (18), etc. In addition, Dan can attenuate hypoxia-induced dyslipidemia (19) and reduce inflammation induced by high fat *in vivo* (20). Increasing evidence has demonstrated that Dan plays protective roles in coronary heart diseases. Interestingly, it was reported that Dan may protect myocardium against IRI *in vivo* (21). However, the functional role and molecular mechanism of Dan in CMs upon IRI was largely not elucidated.

To investigate the mechanism of Dan attenuating acute IRI, we built *in vivo* and *in vitro* models using mice and primary neonatal rat CMs (NRCMs), respectively. For exploring the downstream target and pathway of Dan, pharmacological inhibition strategy was used in this study. To reveal the mechanism of Dan in IRI may extend the knowledge of Chinese medicine and provide new strategy for precise treatment of ischemic heart diseases.

## Materials and Methods

### Animals and IRI Modeling

Male C57BL/6J mice (8 weeks old) were purchased from Charles River (Beijing, China) and were housed in a specific pathogen-free atmosphere. To investigate the effect of Danlou tablet in IRI, Dan was dissolved with saline (70 mg/ml, ultrasonic for 1 h) and was administrated to mice by gavage at a dose of 700 mg/kg/d for 2 consecutive weeks before acute IRI modeling. The control mice were administrated with equal volume of saline. Then, myocardial IRI was induced by ligating the left anterior descending artery for 30 min followed by reperfusion for 24 h according to the previous study (22). To study whether peroxisome proliferator-activated receptor gamma (PPARγ) mediated the function of Dan in IRI, mice were intraperitoneally injected with PPARγ inhibitor T0070907 (Selleck, S2871) at a dose of 1 mg/kg/d in the presence of Dan treatment. All animal experiments were approved by the Ethics Committee of Shanghai University and performed in accordance with the guidelines.

### 2,3,5-Triphenyl Tetrazolium Chloride (TTC) Staining

To evaluate the effect of Dan on the infarct size after acute IRI, TTC staining was performed after 24 h of reperfusion. Briefly, 1 ml of Evans blue (1% in phosphate-buffered saline) was injected into the left ventricle, and the heart was sliced to 1-mm-thick tissue sections and stained with TTC. The homogeneity of modeling was assessed by calculating the ratio of area at risk to left ventricle weight (AAR/LV), and the infarct size of heart was assessed by the ratio of infarct size/area at risk (INF/AAR).

### Primary NRCM Isolation and Treatment

Left ventricles were freshly harvested from neonatal Sprague-Dawley rats (1–3 days old) and minced into 1-mm^2^ small pieces on ice. Primary NRCMs were digested using Collagenase II (Gibco, 17101015) and Pancreatin from porcine pancreas (Sigma, P3292) and isolated using Percoll (GE healthcare, 17-0891-01) centrifugation (23). NRCMs were cultured in Dulbecco's modified Eagle's medium (DMEM) supplemented with 5% fetal bovine serum and 10% horse serum for further experiments. To explore the effect of Dan on CM apoptosis, NRCMs were treated with 50 μg/ml of Dan for 48 h followed by oxygen-glucose deprivation–reperfusion (OGD/R) modeling. To inhibit the activity of PPARγ in NRCMs, two PPARγ inhibitors GW9662 (10 μm, Selleck, S2915) and T0070907 (1 μm, Selleck, S2871) were used to treat NRCMs for 24 h *in vitro*, respectively.

### Oxygen-Glucose Deprivation–Reperfusion

To induce OGD/R model, NRCMs were cultured in serum-free and glucose-deprived DMEM medium under oxygen deprivation atmosphere at 37°C for 8 h. Then, the cells were cultured with NRCM culture medium under normal oxygen condition for 12 h. The OGD/R-driven cells were divided into negative control group (vehicle), Dan group (Dan), GW9662 (or T0070907) group, and Dan+GW9662 (or T0070907) group. The control group was not undergoing OGD/R.

### TUNEL Staining

To reveal CM apoptosis *in vivo* and *in vitro*, TdT-mediated dUTP nick end labeling (TUNEL) staining complemented with α-actinin immunostaining was performed. Briefly, to assess CM apoptosis in mice, heart tissues were harvested after acute IRI for 24 h and embedded into optimal cutting temperature compound (OCT) for subsequent frozen section. The 10-μm-thick heart sections or primary cultured NRCMs were fixed by 4% paraformaldehyde and stained with TUNEL FITC Apoptosis Detection Kit according to the manufacturer's instructions (Vazyme, China). Immunostaining for α-actinin (Sigma, A7811) was performed to label CMs. Finally, sections were incubated with Hoechst for 20 min at room temperature before fluorescence imaging. The percentage of TUNEL-positive CMs was calculated to determine apoptosis in mice hearts upon IRI or in NRCMs upon OGD/R modeling.

### Western Blot

NRCMs or heart tissues were homogenized in RIPA lysis buffer complemented with 1% PMSF for 30 min at 4°C and subsequently centrifuged at 12,000 g for 20 min. Then, protein supernatants were obtained and added with loading buffer to boil for 10 min. A total of 10 to 30 μg proteins were used to perform western blot as previously reported (24). Primary antibodies for Bax (Abclonal, A0207), Bcl-2 (Abclonal, A2845), caspase 3 (Cell Signaling, 9662), and PPARγ (Abclonal, A0270) were used, respectively. GAPDH or β-actin was used as an internal control.

### Reverse Transcription Quantitative PCR

Total RNA was isolated from mouse heart tissues or NRCMs using TRIzol RNAiso Plus Kit (TaKaRa) and then reverse-transcribed to cDNA using RevertAid First Strand cDNA Synthesis Kit (Thermo K1622). The mRNA levels were analyzed by quantitative PCR (qPCR) using TaKaRa SYBR Premix Ex Taq™ (Tli RNaseH Plus, Japan) on Roche LightCycler480 PCR System. The primers used were as follows: mmu-PPARγ (5′-3′ forward and reverse) CGAGAAGGAGAAGCTGTTG and TCAGCGGGAAGGACTTTA; rno-PPARγ (5′-3′ forward and reverse) GGGAGTTCCTCAAAAGCC and TTCACGTTCAGCAAGCC; 18s (5′-3′forward and reverse) TCAAGAACGAAAGTCGGAGG and GGACATCTAAGGGCATCAC. The 18s was used as internal controls.

### Statistical Analysis

All data in this study were analyzed using SPSS software version 20.0 or GraphPad Prism 8.0 software and were reported as mean ± standard deviation (SD). Student's *t*-test (two-sided) was used for two independent group comparisons. One-way ANOVA followed by Bonferroni or Dunnett's T3 test was used for comparisons among 3 groups. Two-way ANOVA followed by Tukey's correction was performed for more than 3 group comparisons. A *p* < 0.05 was considered statistically significant.

## Results

### Dan Protects Against Acute Myocardial IRI *in vivo*

**Figure 1:**
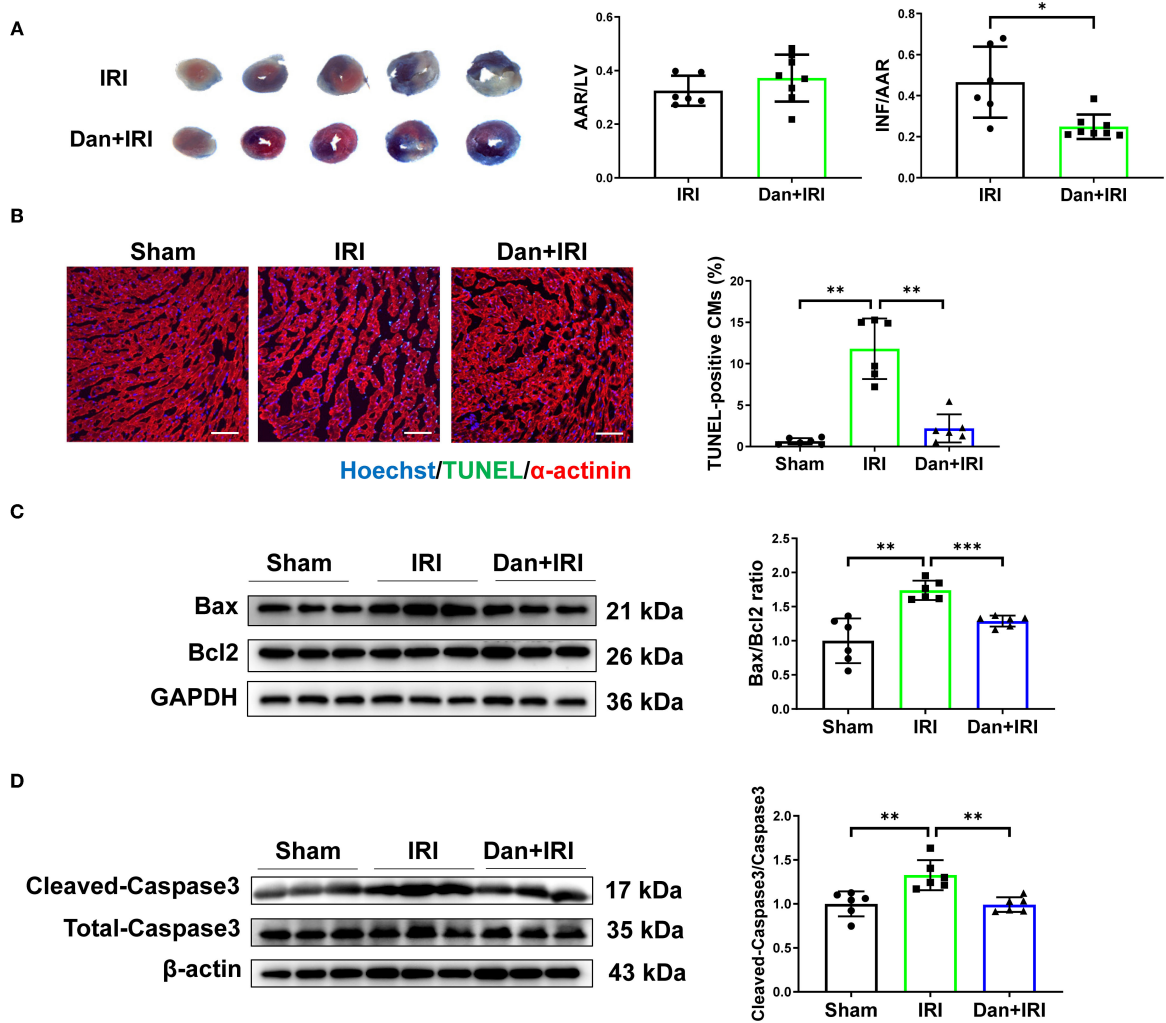
Dan protects mice against acute myocardial ischemia-reperfusion injury *in vivo*. **(A)** Adult male mice were administrated by gavage with 700 mg/kg/d of Dan or equal volume of saline for 2 consecutive weeks followed by acute IRI modeling. TTC staining was performed at 24 h after IRI. The ratio of AAR/LV was determined for the homogeneity of modeling, and the ratio of infarct area/area at risk (INF/AAR) was determined for the infarct size (*n* = 6 vs. 8). **(B)** Representative images and quantification results of TUNEL/α-actinin staining were shown for myocardial apoptosis of mice (*n* = 6). Scale bar = 100 μm. **(C,D)** Western blot analysis for apoptotic-associated proteins in heart tissues, including Bax and Bcl2 **(C)** and cleaved-caspase 3 and total caspase 3 **(D)** (*n* = 6). *p < 0.05; **p < 0.01; ***p < 0.001.

To investigate the potential role of Dan on the heart, mice were undergoing IRI modeling for 24 h followed by TTC staining to evaluate the infarct size. There is no significant difference in AAR/LV ratio between IRI and Dan/IRI group, which demonstrates that a stable IRI modeling was constructed in this study. Interestingly, administration of Dan significantly reduced INF/AAR ratio after acute IRI (Figure 1A), which indicates that Dan protected mice against IRI-induced myocardial injury. To evaluate whether Dan is associated with IRI-induced CM apoptosis, TUNEL staining and apoptotic proteins were assessed in heart tissues. TUNEL/α-actinin staining showed that Dan significantly reduced IRI-induced CM apoptosis (Figure 1B). Consistent with TUNEL data, western blot experiments showed that IRI increased pro-apoptotic markers Bax/Bcl2 ratio and cleaved-caspase 3/caspase 3 ratio, while Dan attenuated their increase induced by IRI (Figures 1C,D). These data provided *in vivo* evidence that Dan exerts a protective effect against acute myocardial IRI and CM apoptosis.

### Dan Attenuates OGD/R-Induced CM Apoptosis

**Figure 2:**
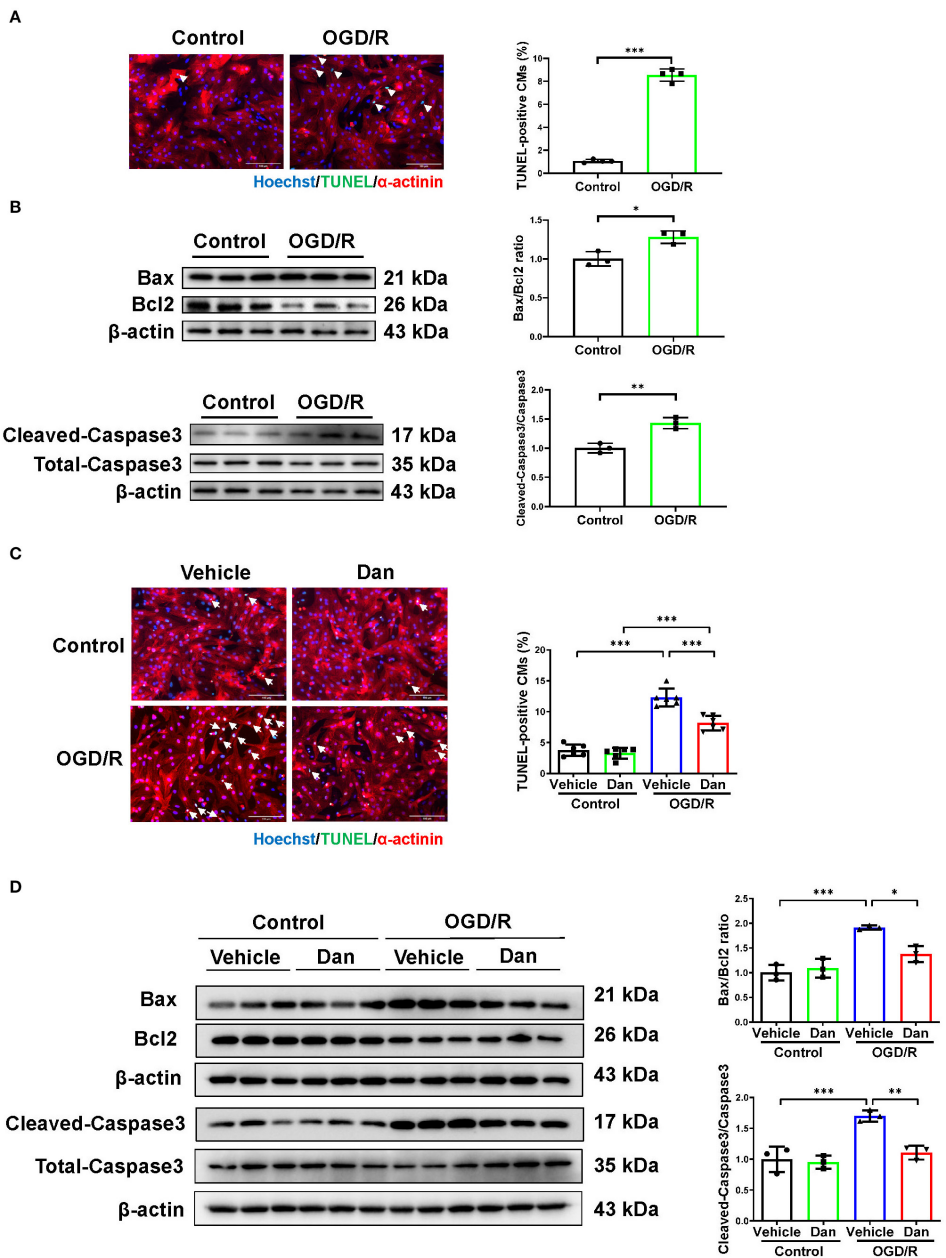
Dan attenuates oxygen-glucose deprivation–reperfusion-induced CM apoptosis *in vitro*. **(A)** Primary NRCMs were submitted to oxygen-glucose deprivation–reperfusion (OGD/R) modeling to induce apoptosis. TUNEL/α-actinin staining was performed to assess CM apoptosis (*n* = 4). Scale bar = 100 μm. **(B)** Western blot analysis for apoptotic-associated proteins in OGD/R-induced apoptosis of NRCMs (*n* = 3). **(C)** TUNEL/α-actinin staining for OGD/R-induced apoptosis of NRCMs in the presence or absence of Dan treatment (*n* = 6). Scale bar = 100 μm. **(D)** Western blot analysis for apoptotic-associated proteins in OGD/R-induced apoptosis of NRCMs in the presence or absence of Dan treatment (*n* = 3). *p < 0.05; **p < 0.01; ***p < 0.001.

To further evaluate the effects of Dan on CM apoptosis *in vitro*, primary NRCMs were isolated and used to mimic IRI *in vitro*. During myocardial ischemia stress, hypoxia stress is usually accompanied with an alteration of glucose metabolism in the myocardium. Thus, we used the deprivation and reperfusion of both oxygen and glucose in cultured CMs *in vitro* in order to better mimic myocardial I/R injury *in vivo* in our study. TUNEL staining and pro-apoptotic protein markers were performed to assess the efficiency of OGD/R modeling. Notably, OGD/R caused significant increase in TUNEL-positive CMs (Figure 2A), and increased Bax/Bcl2 ratio and cleaved-caspase 3/caspase 3 ratio (Figure 2B). These data demonstrated that we successfully constructed OGD/R-induced CM apoptosis model *in vitro*. Then, we treated NRCMs with Dan for 48 h to investigate the functional role of Dan on OGD/R-induced CM apoptosis. Under basal condition, treatment with Dan did not influence CM apoptosis, while it attenuated ODG/R-induced increase in TUNEL-positive CMs (Figure 2C). Western blot showed that Dan treatment did not influence the Bax/Bcl2 ratio or caspase 3 cleavage at baseline, but reduced the Bax/Bcl2 ratio and cleaved-caspase 3/caspase 3 ratio in CMs upon OGD/R stress (Figure 2D). All these data indicate that treatment with Dan can effectively protect CMs against OGD/R-induced apoptosis.

### Dan Prevents CM Apoptosis Through Activating PPARγ

**Figure 3:**
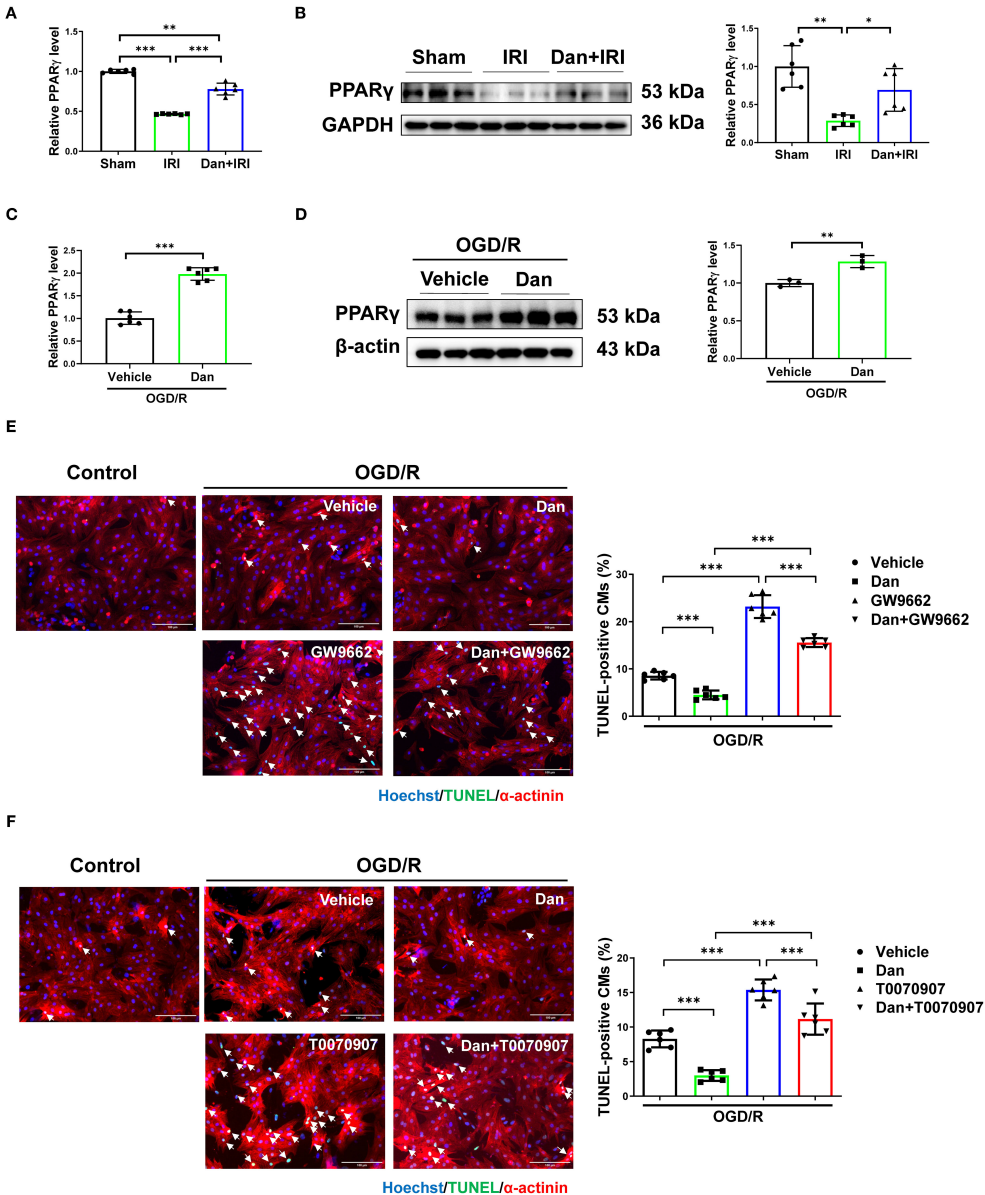
Dan prevents CM apoptosis through activating PPARγ *in vitro*. **(A,B)** RT-qPCR [**(A)**, *n*= 6] and western blot [**(B)**, *n*= 6] for PPARγ in mice heart tissues of myocardial ischemia-reperfusion injury (IRI) in the presence or absence of Dan treatment. **(C,D)** RT-qPCR [**(C)**, *n*= 6] and western blot [**(D)**, *n*= 3] for PPARγ in oxygen-glucose deprivation–reperfusion (OGD/R)-induced apoptosis of NRCMs in the presence or absence of Dan treatment. **(E,F)** TUNEL/α-actinin staining for OGD/R-induced apoptosis of NRCMs treated with PPARγ inhibitors, GW9662 **(E)** or T0070907 **(F)**, in the presence or absence of Dan treatment (*n* = 6). Scale bar = 100 μm. *p < 0.05; **p < 0.01; ***p < 0.001.

Multiple evidence showed that PPARγ was involved in IRI not only in myocardium (25) but also in liver (26). Reduced PPARγ expression or activity is associated with aggravated IRI. To assess whether PPARγ is involved in the cardioprotective roles of Dan after IRI, we first examined PPARγ expression level in heart tissues. We observed that IRI significantly downregulated PPARγ in the heart, while Dan reversed IRI-induced PPARγ reduction at both mRNA and protein level (Figures 3A,B). To further investigate whether PPARγ is involved in Dan-induced cardioprotection, we also assessed PPARγ expression in OGD/R-induced CM apoptosis model and performed functional rescue experiments *in vitro*. Our data showed that Dan treatment caused an increase in PPARγ mRNA and protein levels in NRCMs after OGD/R modeling (Figures 3C,D). To block the activity of PPARγ, we chose two PPARγ inhibitors (GW9662 and T0070907) to observe the change of CM apoptosis in OGD/R model. In OGD/R-induced CM apoptosis model, Dan treatment reduced TUNEL-positive CMs, while Dan co-treatment with GW9662 reversed this change in NRCMs (Figure 3E). In addition, the protective effect of Dan against OGD/R-induced CM apoptosis was also abolished by Dan co-treatment with T0070907 (Figure 3F). These data indicate that PPARγ activation is necessary to mediate the protective effect of Dan against OGD/R-induced CM apoptosis.

### Dan Prevents Acute Myocardial IRI Through Activating PPARγ *in vivo*

**Figure 4:**
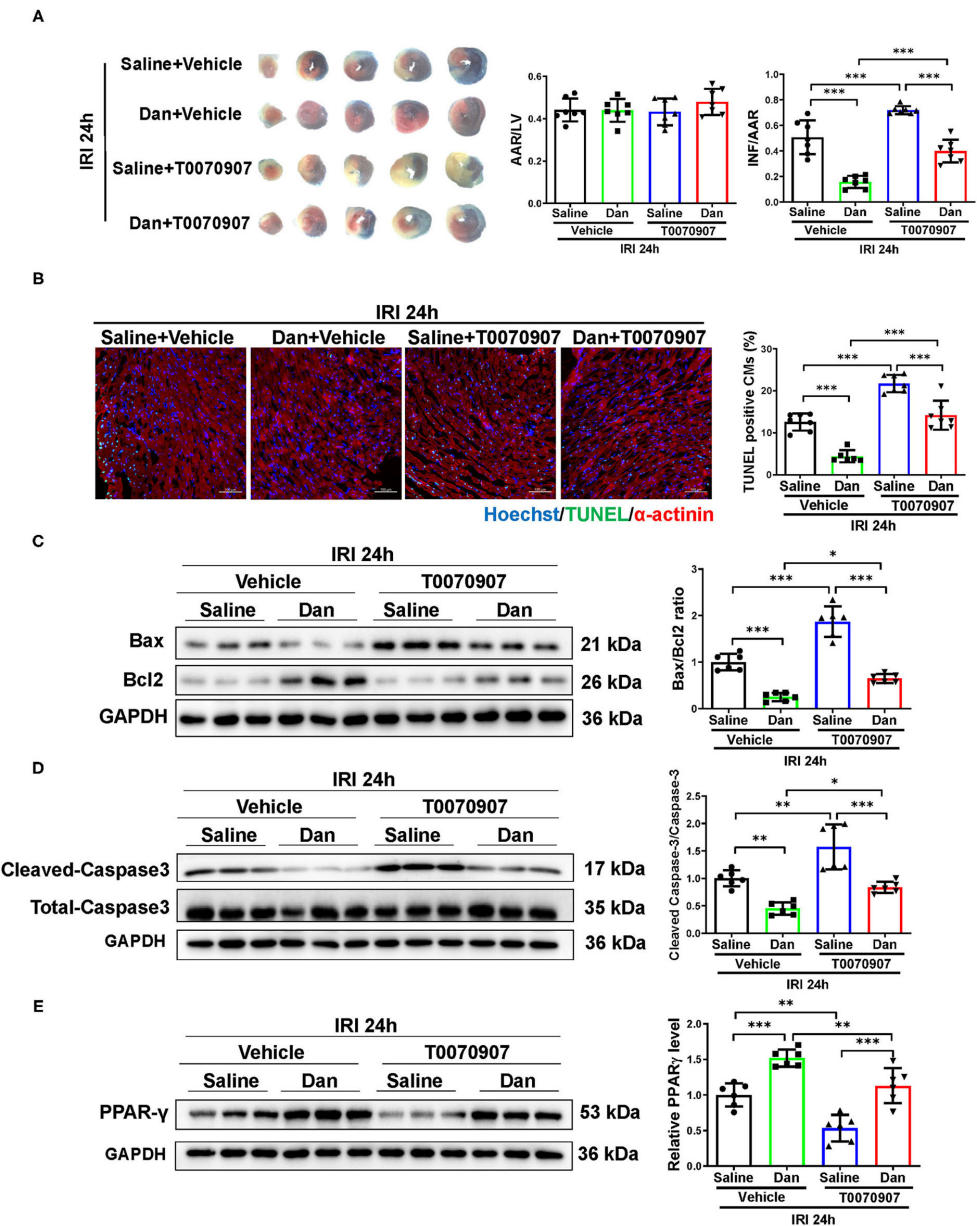
Inhibition of PPARγ attenuates Dan-induced cardioprotection in acute myocardial ischemia-reperfusion injury *in vivo*. **(A)** Adult male mice were intraperitoneally injected with T0070907 (1 mg/kg/day) or vehicle controls and administrated by gavage with 700 mg/kg/d of Dan for 2 consecutive weeks followed by myocardial ischemia-reperfusion injury (IRI) modeling for 24 h. TTC staining was performed at 24 h after IRI. The ratio of AAR/LV was determined for the homogeneity of modeling, and the ratio of infarct area/area at risk (INF/AAR) was determined for the infarct size (*n* = 7). **(B)** Representative images and quantification results of TUNEL/α-actinin staining were shown for myocardial apoptosis of mice (*n* = 6–7). Scale bar = 100 μm. **(C,D)** Western blot analysis for apoptotic-associated proteins in heart tissues, including Bax and Bcl2 **(C)** and cleaved-caspase 3 and total caspase 3 **(D)** (*n* = 6). **(E)** Western blot for PPARγ in mice IRI heart tissues administrated with T0070907 or vehicle in the presence or absence of Dan treatment (*n* = 6). *p < 0.05; **p < 0.01; ***p < 0.001.

To further investigate whether PPARγ mediates the cardioprotective effects of Dan *in vivo*, we performed functional rescue experiments using PPARγ inhibitor T0070907 in Dan-treated IRI mice. Mice were randomly arranged into four groups, which include vehicle+IRI, Dan+IRI, T0070907+IRI, and Dan+T0070907+IRI. TTC staining was performed to evaluate the infarct size after acute IRI modeling for 24 h. As shown in Figure 4A, Dan effectively reduced the INF/AAR ratio compared to vehicle-treated IRI group. Interestingly, co-treatment with Dan and T0070907 attenuated the protective effect of Dan in reducing the infarct size in IRI mice (Figure 4A). Next, we also performed apoptotic analysis *via* TUNEL staining and western blot. Compared to Dan-treated IRI group, co-treatment with Dan and T0070907 caused increased TUNEL-positive CMs (Figure 4B), as well as increased Bax/Bcl2 ratio and cleaved-caspase 3/caspase 3 ratio (Figures 4C,D). Consistent with these results, Dan treatment was able to increase PPARγ protein level in heart tissues, while PPARγ inhibitor T0070907 attenuated Dan-induced PPARγ expression (Figure 4E). These data demonstrate that PPARγ activation is also necessary to mediate the protective effect of Dan against acute IRI and myocardial apoptosis *in vivo*.

## Discussion

**Figure 5:**
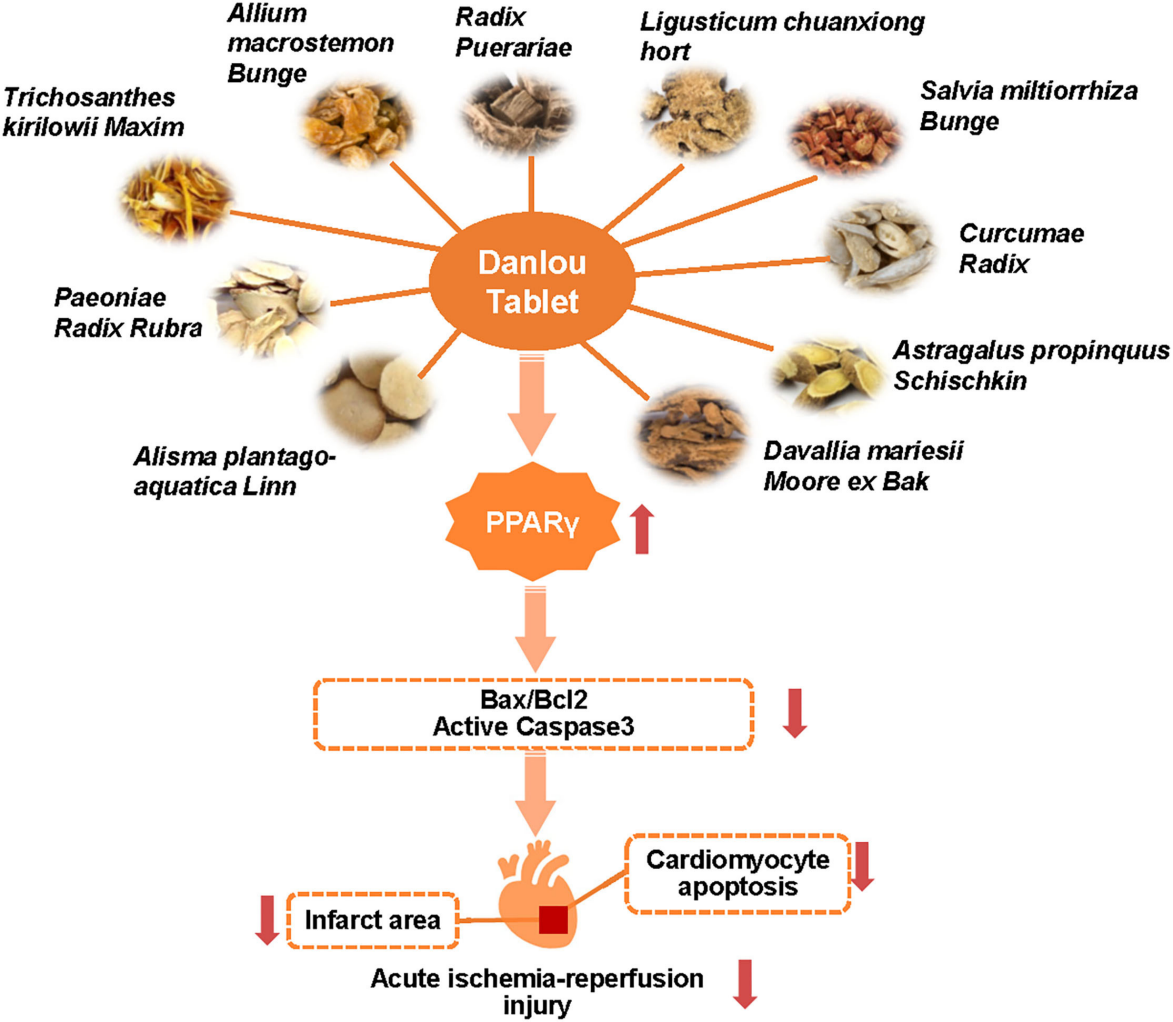
Danlou tablet protects against acute myocardial ischemia-reperfusion injury and reduces CM apoptosis through activating PPARγ.

In this study, we explored the potential role of Dan in acute cardiac IRI and myocardial apoptosis and further investigated the mechanism of Dan-induced cardioprotection. Our findings reveal that Dan protects against acute cardiac IRI and myocardial apoptosis, while PPARγ inhibition prior to IRI attenuates Dan-induced cardioprotection *in vivo* and *in vitro*, which demonstrates that Dan prevents acute myocardial IRI through activating PPARγ (Figure 5). We revealed the mechanisms of Dan-induced cardioprotective role in IRI, which may extend our knowledge of Dan in reducing IRI and provide a potential strategy for IRI treatment.

Dan is a complex with ten kinds of ingredients, which includes *Allium macrostemon Bunge, Radix Puerariae, Salvia miltiorrhiza Bunge*, and *Paeoniae Radix Rubra*, etc. Increasing evidence has shown that Dan plays important roles in improving coronary heart diseases (27), such as atherosclerosis (18) and myocardial injury (17). However, the mechanisms of Dan-induced cardioprotection need to be illustrated. To date, some studies showed that Dan can attenuate oxidative redox state and inflammatory reaction to regulate cardiac homeostasis. For example, a recent study reported that Dan significantly improved chronic stable angina through reducing circulating inflammatory factors (interleukin-6/IL-6, interleukin-10/IL-10, and tumor necrosis factor-α/TNF-α, etc.) and regulating gut microbiota (28). Another study showed that Dan treatment inhibited inflammation in high-fat diet-induced atherosclerosis *via* suppressing nuclear factor kappa-B (NF-κB) signaling pathway (20). Furthermore, administration of Dan reduced infarct area through inducing endothelial and inducible nitric oxide synthase production in rat model (29). Of interest, the involvement of PPARγ in Dan-induced cardioprotection has not been investigated in the past years.

In this study, we first demonstrated that Dan had protective effect against acute cardiac IRI. We provided direct evidence that Dan could attenuate CM apoptosis both *in vivo* and *in vitro*. We further revealed that Dan treatment can activate PPARγ in the IRI hearts and OGD/R-stressed CMs. Using PPARγ inhibitors *in vivo* and *in vitro*, we demonstrated that PPARγ inhibition attenuated the protective effect of Dan in acute cardiac IRI and CM apoptosis, which indicates that PPARγ was involved in the cardioprotection of Dan upon IRI. These data suggest that Dan may be a potential activator of PPARγ, whose usage may deserve further investigations in other PPARγ-associated diseases.

Peroxisome proliferator-activated receptor gamma, an important transcription factor, is involved in multiple physiological and pathological processes such as cell differentiation, glucose–lipid metabolism, and endothelial function (30). Increasing evidence has revealed the effect of PPARγ in CVDs. For example, PPARγ was found to be involved in doxorubicin (Dox)-induced acute cardiac injury in mice, whose inactivity blocked miR-128-3p inhibition-triggered protection upon Dox injury (31). Inhibiting PPARγ by GW9662 can abolish piperine-induced cardioprotection in cardiac fibrosis model (32). Furthermore, PPARγ has been proved to be beneficial for the heart, whose activation or upregulation can attenuate diabetic cardiomyopathy (33), atherosclerosis (34), hypertension (35), and heart failure (33, 36). It is widely accepted that aerobic exercise is protective for the heart (37, 38). Of note, PPARγ is also involved in exercise-induced cardioprotection (39). Therefore, PPARγ is considered as a therapeutic target in CVDs such as atherosclerosis and heart failure, and its activators have been tried to be used as potential strategy for CVD treatment. For example, rosiglitazone (RGZ) has been widely used in type 2 diabetes therapy (30). However, whether and how PPARγ activation can be applicated to treat cardiovascular-related diseases need more clinical trials.

In conclusion, our findings reveal that Dan can protect myocardial tissue against acute IRI *via* activating PPARγ *in vivo* and *in vitro* and demonstrate that Dan is a potential activator of PPARγ in reducing myocardial apoptosis. This study may extend our knowledge of Chinese medicine and provide new strategy for the precise treatment of ischemic heart diseases.

## Data Availability Statement

The raw data supporting the conclusions of this article will be made available by the authors, without undue reservation.

## Ethics Statement

The animal study was reviewed and approved by the Ethics Committee of Shanghai University.

## Author Contributions

MW, MG, XM, and LL performed the experiments and analyzed the data. MZ provided technical assistance. YB and HW designed the study and drafted the manuscript. All authors read and approved the final manuscript.

## Funding

This work was supported by the grants from National Key Research and Development Program of China (2017YFC1700401 to MZ and YB), National Natural Science Foundation of China (81970335 and 82170285 to YB, 82000253 to HW), Shanghai Rising-Star Program (19QA1403900 to YB), Shanghai Committee of Science and Technology (21SQBS00100 to YB), and Chenguang Program of Shanghai Education Development Foundation and Shanghai Municipal Education Commission (20CG46 to HW).

## Conflict of Interest

The authors declare that the research was conducted in the absence of any commercial or financial relationships that could be construed as a potential conflict of interest.

## Publisher's Note

All claims expressed in this article are solely those of the authors and do not necessarily represent those of their affiliated organizations, or those of the publisher, the editors and the reviewers. Any product that may be evaluated in this article, or claim that may be made by its manufacturer, is not guaranteed or endorsed by the publisher.
